# Physiological response to elevated temperature and *p*CO_2_ varies across four Pacific coral species: Understanding the unique host+symbiont response

**DOI:** 10.1038/srep18371

**Published:** 2015-12-16

**Authors:** Kenneth D. Hoadley, D. Tye Pettay, Andréa G. Grottoli, Wei-Jun Cai, Todd F. Melman, Verena Schoepf, Xinping Hu, Qian Li, Hui Xu, Yongchen Wang, Yohei Matsui, Justin H. Baumann, Mark E. Warner

**Affiliations:** 1 School of Marine Science and Policy, University of Delaware, Lewes, DE, United States; 2 School of Earth Sciences, The Ohio State University, Columbus, OH, United States; 3 Department of Marine Sciences, University of Georgia, Athens, GA, United States; 4 Reef Systems Coral Farm, New Albany, OH, United States

## Abstract

The physiological response to individual and combined stressors of elevated temperature and *p*CO_2_ were measured over a 24-day period in four Pacific corals and their respective symbionts *(Acropora millepora/Symbiodinium C21a, Pocillopora damicornis/Symbiodinium C1c-d-t, Montipora monasteriata/Symbiodinium C15*, and *Turbinaria reniformis/Symbiodinium trenchii*). Multivariate analyses indicated that elevated temperature played a greater role in altering physiological response, with the greatest degree of change occurring within *M. monasteriata* and *T. reniformis.* Algal cellular volume, protein, and lipid content all increased for *M. monasteriata*. Likewise, *S. trenchii* volume and protein content in *T. reniformis* also increased with temperature. Despite decreases in maximal photochemical efficiency, few changes in biochemical composition (i.e. lipids, proteins, and carbohydrates) or cellular volume occurred at high temperature in the two thermally sensitive symbionts *C21a* and *C1c-d-t*. Intracellular carbonic anhydrase transcript abundance increased with temperature in *A. millepora* but not in *P. damicornis*, possibly reflecting differences in host mitigated carbon supply during thermal stress. Importantly, our results show that the host and symbiont response to climate change differs considerably across species and that greater physiological plasticity in response to elevated temperature may be an important strategy distinguishing thermally tolerant vs. thermally sensitive species.

Coral reefs represent one of the most biologically rich ecosystems on the planet. The importance of scleractinian corals to continual reef accretion has placed much attention on understanding how they will respond to future climate conditions, including elevated seawater temperature and ocean acidification. Water temperatures of just a few degrees above the summer maximum average can lead to large-scale coral bleaching events^1^, which are often characterized by the expulsion of symbiotic dinoflagellates (*Symbiodinium* spp.). Such bleaching events can result in high coral mortality and a significant loss in coral cover^1^.

Photoinactivation and damage to the photosystem II (PSII) reaction center is often a first sign of temperature stress within thermally susceptible *Symbiodinium*^2^,^3^. Photoinactivation can result in reduced photosynthetic rates and elevated reactive oxygen species, further damaging the symbiont and coral^4^,^5^,^6^. This stress can change the energetic/metabolic demands of the symbiont, reducing the amount of photosynthate translocated to the host. In turn, host catabolic pathways are utilized to supply additional energy to compensate for the loss of translocated carbon and/or to keep pace with greater metabolic demand from high temperature stress, leading to a decline in one or more of the host’s energy reserves of proteins, carbohydrates and lipids (defined hereafter as the biochemical composition)^7^,^8^,^9^. However, other mechanisms such as enhanced heterotrophy can compensate for reduced photosynthate translocation, and maintain biochemical composition^8^,^10^,^11^. Similar to other marine phytoplankton, symbiont biochemical composition may also change in response to temperature stress^12^,^13^. Thus, due to variation in host and symbiont thermal tolerance, the overall thermal response of the holobiont (i.e. the host + the symbiont) can vary widely across different corals.

Ocean acidification (abbreviated OA hereafter), which describes the decrease in seawater pH resulting from increasing atmospheric CO_2_ partial pressure (*p*CO_2_) levels and subsequent dissolution and acid production, has the potential to affect many aspects of coral physiology. Several studies have noted reduced coral calcification at high *p*CO_2_ (e.g.,^11^,^14^,^15^). However, some corals show no decline or a delayed decline in calcification well below the current aragonite saturation state, suggesting the response to high *p*CO_2_ is highly species specific (e.g.,^11^,^14^,^16^). Compared to calcification studies, less attention has been placed on other aspects of holobiont physiology, including primary productivity and biochemical composition. As CO_2(aq)_ concentrations increase with OA, symbiont productivity could increase due to a release from carbon limitation^17^. Greater net productivity with elevated CO_2_ was reported for symbiotic sea anemones in laboratory experiments and near natural CO_2_ seeps^18^,^19^. Highly variable, CO_2_ driven changes in respiration and productivity may result in significant changes to host and symbiont metabolism, and are likely to affect the overall health and resilience of the coral as these organisms cope with additional environmental stressors.

High CO_2_ conditions enhanced productivity for some cultured *Symbiodinium*, but not others^20^, and this variability may be linked with differences in carbon acquisition as well as the preference in dissolved inorganic carbon (CO_2_ vs. HCO_3_^-^) among algal species^21^. In addition, increased respiration rates, along with changes in the transcriptional response of genes involved with metabolic pathways and cellular structure, were noted in scleractinians in response to OA^22^,^23^. Specifically, genes associated with energy production and ion transport were up-regulated under acidification conditions^24^, suggesting changes in the biochemical composition of the symbiosis.

Research to date has largely focused on the physiological changes and metabolic pathways most important towards understanding the coral response to climate change. However, because most studies have focused on just a few model scleractinian species^25^,^26^,^27^,^28^,^29^, less is known about the potential for physiological diversity within each unique host/symbiont combination. Whether or not the physiological responses to both elevated temperature and *p*CO_2_ that have been described^11^,^30^,^31^ accurately depict the range of coral responses within the entire Scleractinian taxa remains to be seen.

Here, we utilized a combination of physiological, and transcriptional approaches to characterize both the host and symbiont response to elevated temperature and *p*CO_2_. Through the comparative analysis of multiple physiological variables within the host and symbiont, the unique response to temperature and *p*CO_2_ within each species was characterized. This approach highlights the diversity of physiological responses within scleractinian corals and how each host/symbiont system responds uniquely to a changing environment anticipated under future climate change conditions.

## Results

### Symbiont Identification

Continual specificity between hosts and symbionts was noted for the duration of the experiment, with *Symbiodinium C21a* in *A. millepora, C1c-d-t* in *P. damicornis, C15* in *M. monasteriata*, and *S. trenchii* (formerly called *D1a*) in *T. reniformis.* No other symbiont fingerprints were detected.

### Multivariate Analyses (ANOSIM and SIMPER)

**Figure 1:**
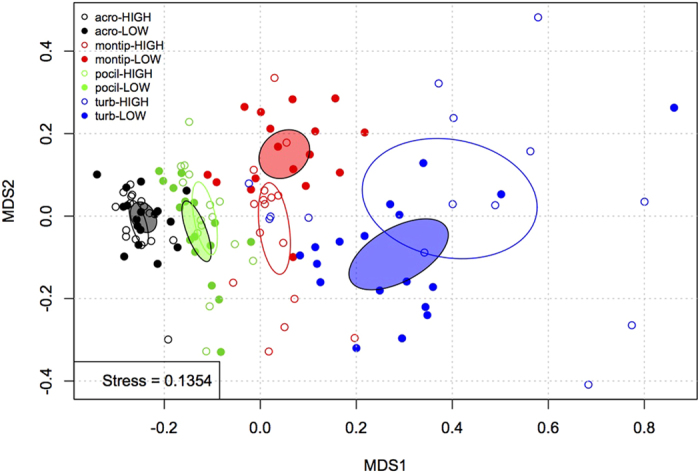
Non-metric multidimensional scaling (nMDS) plot displaying similarities within temperature treatments for the four coral species. Black = *A*. *millepora*, green = *P*. *damicornis*, red = *M*. *monsateriata* and blue = *T. reniformis.* Closed circles represent low temperature treatments and open circles represent high temperature treatments. Ellipses represent a 99% confidence bubble around the mean for low temperature (closed ellipse) and high temperature (open ellipse) treatments. Because the ANOSIM analysis found CO_2_ to be insignificant or explain only minimal separation across fragments, only temperature differences are depicted in this figure.

The greatest separation between low and high temperature treatments was found within *M. monasteriata*, followed by *T. reniformis* and then *A. millepora* (Table. 1). Although separation between temperature treatments was also significant for *P. damicornis*, the correlation was very low (r < 0.2), suggesting only a minimal thermal effect within this species. The relative contribution of the measured variables to the overall dissimilarity between low and high temperature differed among species, with host lipid and protein and LEDR contributing 36% in *A. millepora* whereas symbiont lipids and LEDR contributed the most (30%) in *M. monasteriata.* Lastly, temperature induced changes in *T. reniformis* were mostly explained through changes in symbiont protein and lipid content, as well as LEDR, accounting for 43% of the dissimilarity (Section S9). Significant CO_2_ effects were only observed for *A. millepora* and *T. reniformis* (Table 1). However, correlation values for CO_2_ in both *A. millepora* and *T. reniformis* were very low and therefore were not followed by SIMPER analysis. Figure 1 provides an overview of the above trends. (Fig. 1).

### Photosynthesis and Respiration

**Figure 2:**
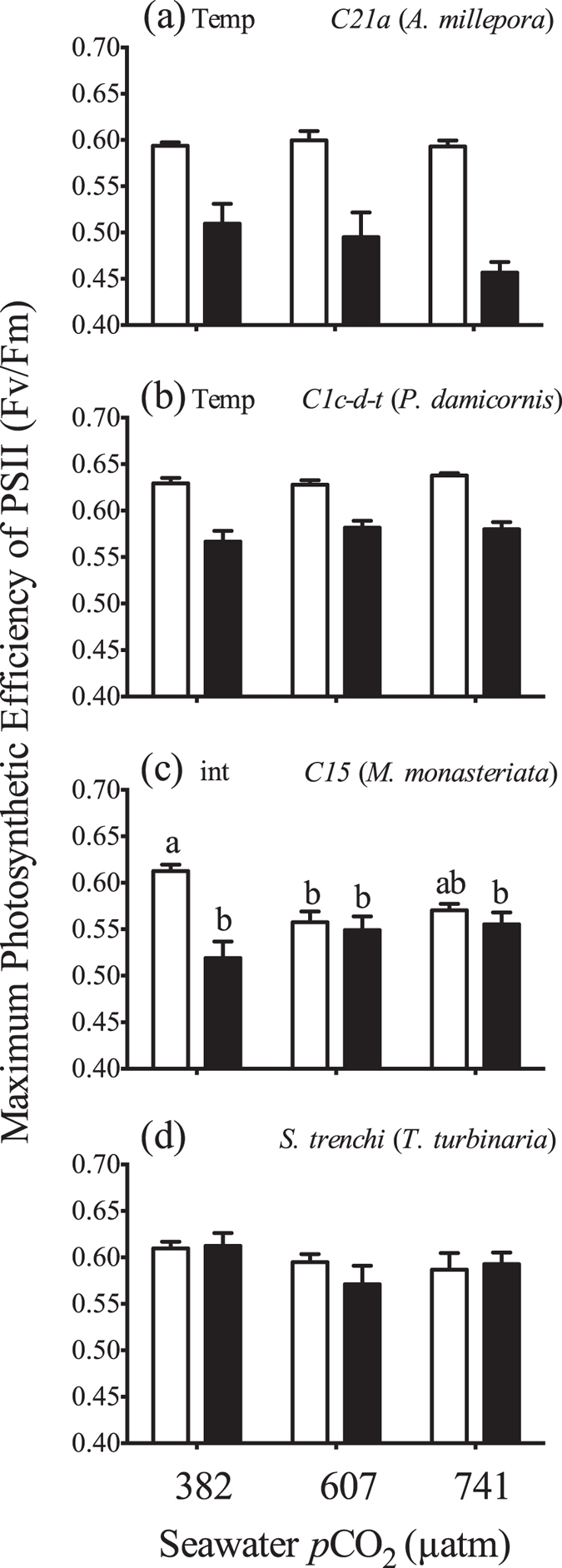
Average (±1SE) maximum photosynthetic efficiency of PSII (F_v_/F_m_) in *Acropora millepora* (a), *Pocillopora damicornis* (b), *Montipora monasteriata* (c), and *Turbinaria reniformis* (d) at three *p*CO_2_ levels and 26.5 °C (light bars) or 31.5 °C (dark bars). For each pane, the designations ‘temp’, ‘*p*CO_2_’ and ‘int’ indicate significant temperature, *p*CO_2_ or interactive effects (two-way ANOVA). If a *p*CO_2_ effect was observed, the letters indicate significant differences between *p*CO_2_ groups (n = 6). If an interactive effect was observed, the letters above each bar indicate significant differences among the 6 treatments.

**Figure 3:**
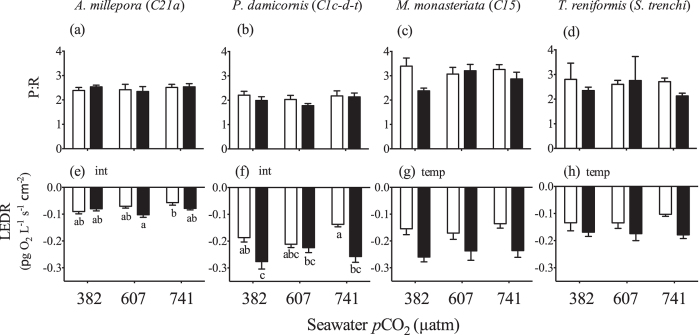
Average (±1SE) photosynthesis to respiration (P:R) and light enhanced dark respiration (LEDR) of *Acropora millepora* (a,e), *Pocillopora damicornis* (b,f)*, Montipora monasteriata* (c,g), and *Turbinaria reniformis* (d,h) at three *p*CO_2_ levels and 26.5 °C (light bars) or 31.5 °C (dark bars). For each pane, the designations ‘temp’, ‘*p*CO_2_’ and ‘int’ indicate significant temperature, *p*CO_2_ or interactive effects (two-way ANOVA). If a *p*CO_2_ effect was observed, the letters indicate significant differences between *p*CO_2_ groups (n = 6). If an interactive effect was observed, the letters above each bar indicate significant differences among the 6 treatments.

Elevated temperature significantly reduced maximum PSII photosynthetic efficiency (F_v_/F_m_) in both *C21a* in *A. millepora* and *C1c-d-t in P. damicornis* (Fig. 2a,b) (Supplemental Section S3). Significant interactive effects were observed for *C15* in *M. monasteriata*, as F_v_/F_m_ decreased with temperature only within the ambient *p*CO_2_ treatment (Fig. 2c) (Table S3). The control treatment was also significantly higher than medium *p*CO_2_ treatments and the high temperature, high *p*CO_2_ treatment as well. F_v_/F_m_ did not change for *S. trenchii* in *T. reniformis* (Fig. 2d). There was no change in the photosynthesis to respiration ratios (P:R) across treatments for any coral species (Fig. 3a–d) (Section S4). For *A. millepora, a* significant interactive effect was observed for light enhanced dark respiration (LEDR). However, pair-wise comparisons revealed significant differences only between the high temperature, medium *p*CO_2_ treatment and the ambient temperature, high *p*CO_2_ treatment (Fig. 3e). For *P. damicornis*, significant interactive effects were also observed, as LEDR increased with temperature but was significant only within the low and high *p*CO_2_ treatments (Fig. 3f). Elevated temperature also significantly increased LEDR in *M. monasteriata*, and *T. reniformis* (Fig. 3g,h).

### Patterns of Symbiont Soluble Proteins, Carbohydrates, Lipids, and Cell  Volume

**Figure 4:**
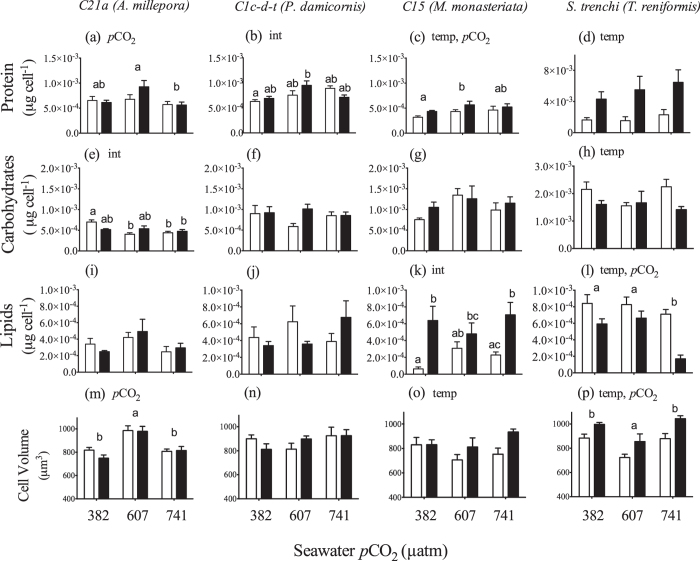
Average (±1SE) total (μg cell^−1^) protein, carbohydrates and lipid content and cell volume for symbiont type *C21a*-*A. millepora* (a,e,i,m), *C1*-*P. damicornis* (b,f,j,n), *C15-M. monasteriata* (c,g,k,o), and *S. trenchii-T. reniformis* (d,h,l,p) at three *p*CO_2_ levels and 26.5 °C (light bars) or 31.5 °C (dark bars). For each pane, the designations ‘temp’, ‘*p*CO_2_’ and ‘int’ indicate significant temperature, *p*CO_2_ or interactive effects (two-way ANOVA). If a *p*CO_2_ effect was observed, the letters indicate significant differences between *p*CO_2_ groups (n = 6). If an interactive effect was observed, the letters above each bar indicate significant differences among the 6 treatments.

Symbiont protein concentration differed with CO_2_ for all three clade C symbionts (Fig. 4a–c), significantly increasing between ambient and medium *p*CO_2_ treatments in *C15* and decreasing between medium and high *p*CO_2_ treatments in *C21a* (Section S5). An interactive effect was noted for *C1c-d-t*, as protein content was significantly elevated within the high temperature, medium CO_2_ treatment as compared to the ambient temperature, low CO_2_ treatment. Notably, protein concentration increased significantly in *S. trenchii* under elevated temperature by an average of 297% (Fig. 4d) whereas a smaller (25%) increase was noted for *Symbiodinium C15* (Section S5).

While there were no changes in carbohydrates for *C1c-d-t* and *C15* symbionts, an interactive effect was observed for *C21a*, as carbohydrates decreased between ambient and medium *p*CO_2_ only within the ambient temperature treatments (Fig. 4e–g). Overall carbohydrates in *S. trenchii* dropped significantly with elevated temperature (Fig. 4d) (Section S5).

Lipid concentrations did not change significantly in *Symbiodinium C21a* or *C1c-d-t* (Fig. 4I,j). However, an interactive effect was observed for symbiont *C15* (), as lipid concentrations increased with temperature but only within the low and high *p*CO_2_ treatments (Fig. 4k). For *S. trenchii*, lipid concentration declined with both temperature and *p*CO_2_ with the high *p*CO_2_ treatments significantly lower than the low and medium *p*CO_2_ treatments (Fig. 4l).

Cell volume of the *C21a* symbiont within the medium *p*CO_2_ treatments was significantly elevated over both ambient and high *p*CO_2_ treatments (Fig. 4m). No significant changes in volume were detected for the *C1c-d-t* symbiont (Fig. 4n). Cell volume increased significantly with temperature for both *C15* (and *S. trenchii* (Fig. 4o,p). For *S. trenchii*, cell volume also varied with *p*CO_2_, as cells within the medium *p*CO_2_ treatment were significantly smaller than those within the low and high *p*CO_2_ treatments (Section S5).

### Patterns of Host Soluble Protein, Carbohydrate and Lipid Content

**Figure 5:**
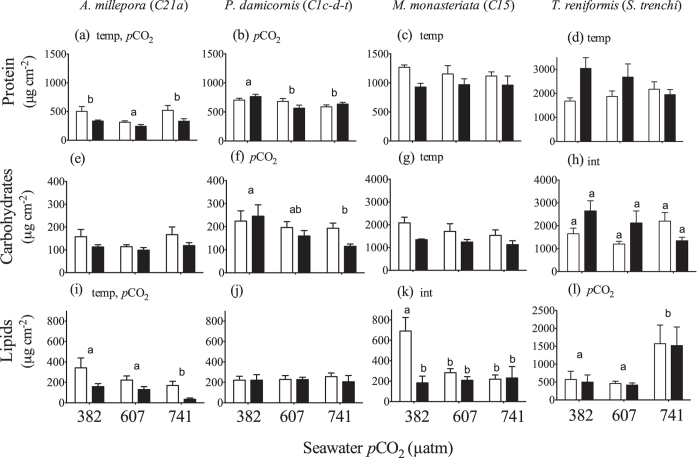
Average (±1SE) total (μg cm^2^) animal protein, carbohydrates and lipid from the host corals *Acropora millepora* (a,e,i), *Pocillopora damicornis* (b,f,j), *Montipora monasteriata* (c,g,k), and Turbinaria reniformis (d,h,l) at three *p*CO_2_ levels and 26.5 °C (light bars) or 31.5 °C (dark bars). For each pane, the designations ‘temp’, ‘*p*CO_2_’ and ‘int’ indicate significant temperature, *p*CO_2_ or interactive effects (two-way ANOVA). If a *p*CO_2_ effect was observed, the letters indicate significant differences between *p*CO_2_ groups (n = 6). If an interactive effect was observed, the letters above each bar indicate significant differences among the 6 treatments.

Host protein concentrations declined with temperature in *A. millepora* and *M. monasteriata*. In contrast, host protein generally increased with temperature in *T. reniformis* (Fig. 5a,c,d; Section S6). In addition, *A. millepora* protein content was significantly lower within the medium *p*CO_2_ treatments as compared to both low and high *p*CO_2_ treatments. Host protein content decreased with increasing *p*CO_2_ for *P. damicornis* and medium and high *p*CO_2_ treatments were significantly lower than ambient *p*CO_2_ (Fig. 5b).

*A. millepora* carbohydrates did not change (Fig. 5e). However, *P. damicornis* carbohydrates decreased with elevated *p*CO_2_ (Fig. 5f), while carbohydrates decreased with temperature in *M. monasteriata* (Fig. 5g). A significant interactive effect was observed for *T. reniformis*, however post hoc analyses found no significant differences among any of the *p*CO_2_ treatments (Fig. 5e) (Section S6).

*A. millepora* lipids decreased significantly as temperature and *p*CO_2_ increased (Fig. 5i). Lipid content did not change significantly in *P. damicornis* (Fig. 5j). Significant interactive effects were observed for *M. monasteriata*, as lipid content was significantly higher within the control temperature and CO_2_ treatment as compared to all other treatments (Fig. 5k). For *T. reniformis*, lipid content was significantly higher in the high *p*CO_2_ treatments as compared to the medium and low *p*CO_2_ treatments (Fig. 5l) (Section S6).

### Gene Expression Patterns in Hosts and Symbionts:

**Figure 6:**
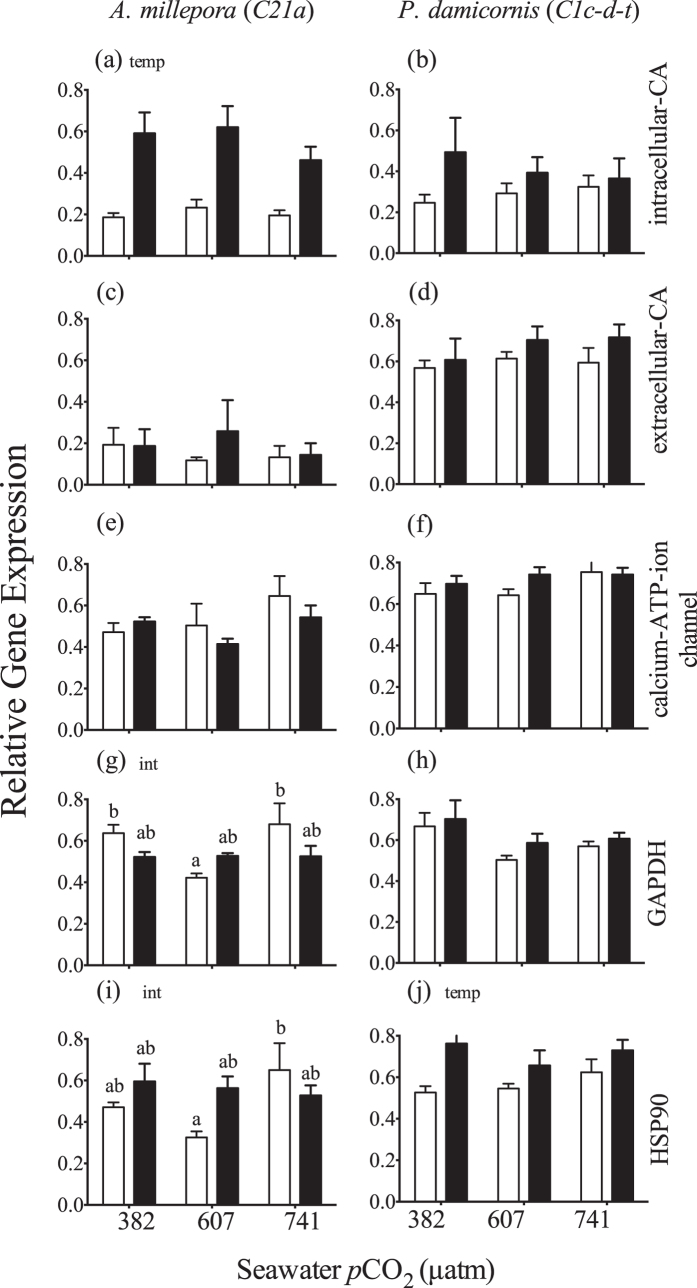
Average (±1SE) relative expression of genes in *A. millepora* and *P. damincornis* encoding intercellular carbonic anhydrase (a,b), extracellular carbonic anhydrase (c,d), calcium ATP ion channel (e,f), glyceraldehyde 3-phosphate dehydrogenase (g,h), and heat shock protein 90 (i,j) at three *p*CO_2_ levels and 26.5 °C (light bars) or 31.5 °C (dark bars). For each pane, the designations ‘temp’, ‘*p*CO_2_’ and ‘int’ indicate significant temperature, *p*CO_2_ or interactive effects (two-way ANOVA). If a *p*CO_2_ effect was observed, the letters indicate significant differences between *p*CO_2_ groups (n = 6). If an interactive effect was observed, the letters above each bar indicate significant differences among the 6 treatments.

*Intracellular carbonic anhydrase* transcript abundance was significantly up-regulated at high temperature in *A. millepora* (Fig 6a; Section S7). In contrast, no significant differences were observed for *P. damicornis* (Fig 6b) or for *extracellular carbonic anhydrase* and *Calcium-ATPase* in either species (Fig. 6c–f) (Section S7). There was a significant interaction for *GAPDH* in *A. millepora* and transcript abundance was significantly higher within both the low and high *p*CO_2_ treatments when compared to the medium *p*CO_2_ treatment, but only within the ambient temperature treatments (Fig. 6g). An interactive effect was also observed for *HSP90* where transcript abundance was significantly higher within the high *p*CO_2_ treatment compared to the medium *p*CO_2_, but only within the ambient temperature treatments (Fig. 6i) (Section S7). There was no change in the GAPDH transcript abundance for *P. damicornis* (Fig. 6h). However there was a significant increase in *HSP90* transcript abundance with temperature (Fig. 6j).

**Figure 7:**
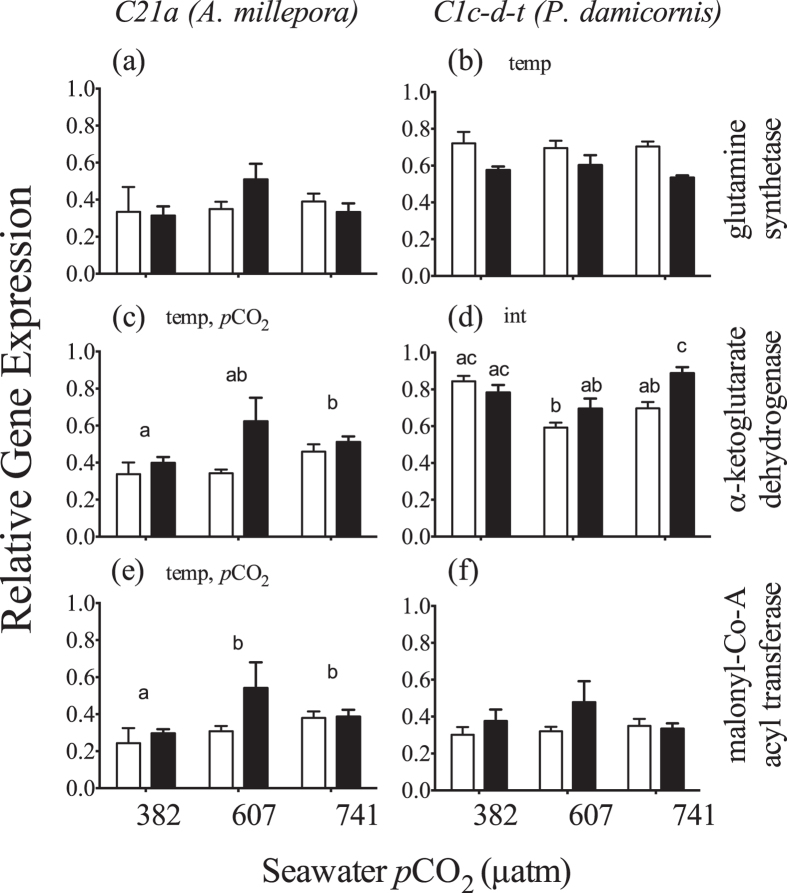
Average (±1SE) relative expression of genes within *Symbiodinium C21a* and *C1c-d-t* encoding glutamine synthetase (a,b), α-ketoglutarate dehydrogenase (c,d), malonyl Co-A acyl transferase (e,f) at three *p*CO_2_ levels and 26.5 °C (light bars) or 31.5 °C (dark bars). For each pane, the designations ‘temp’, ‘*p*CO_2_’ and ‘int’ indicate significant temperature, *p*CO_2_ or interactive effects (two-way ANOVA). If a *p*CO_2_ effect was observed, the letters indicate significant differences between *p*CO_2_ groups (n = 6). If an interactive effect was observed, the letters above each bar indicate significant differences among the 6 treatments.

There was no significant difference in *glutamine synthetase* gene expression for the *C21a* symbiont, whereas transcript abundance decreased with elevated temperature for *C1c-d-t* (Fig. 7a,b). High *p*CO_2_ exposure led to a significant up-regulation in symbiont *C21a* α*-ketoglutarate dehydrogenase* transcript abundance (Fig. 7c). Elevated temperature also increased *C21a α-ketoglutarate dehydrogenase* transcript abundance. However this thermal increase was principally driven by the increase observed under medium *p*CO_2_ (Fig. 7c). There was a significant interactive effect for α*-ketoglutarate dehydrogenase* (Section S8) in *Symbiodinium C1c-d-t* in *P. damicornis*, as transcript abundance increased significantly with temperature but only within the high *p*CO_2_ treatment (Fig. 7d). For the *C21a* symbiont, *malonyl Co-A acyl transferase* increased with *p*CO_2_ and both medium and high *p*CO_2_ treatments were significantly elevated over low *p*CO_2_ (Fig. 7e). Elevated temperature also significantly increased *malonyl Co-A acyl transferase* in this symbiont, yet again, this was principally driven by the increase observed under medium *p*CO_2_ (Fig. 7e). M*alonyl Co-A acyl transferase* expression did not change in the *C1* symbiont (Fig. 7f, Section S8).

## Discussion

The consequence of both host and alga in forming a physiologically unique symbiosis is becoming increasingly clear as we gain a better understanding of the extensive genetic and physiological diversity that exists for both partners. Multivariate analyses utilized here show clear separation among coral species and a more definitive thermal response as compared with *p*CO_2_. Importantly, the largest thermal separation occurred within species hosting the more historically thermally tolerant symbiont types: *Symbiodinium C15* (*M. monasteriata*) and *S. trenchii* (*T. reniformis*)^7^,^32^ indicating larger physiological changes in response to elevated temperature. Assessing thermal sensitivity in corals has largely relied on stability of physiological measurements such as cellular density, chlorophyll and Fv/Fm at elevated temperatures to define thermal tolerance^3^. However, by incorporating a broad range of both host and symbiont physiological variables into our analyses, we show that physiological plasticity may be an important thermal stress mechanism, enabling high temperature tolerance in certain host-symbiont combinations. Additionally, because the direction of thermal separation within *M, monasteriata* and *T. reniformis* differed (Fig 1), the specific holobiont response to high temperature also differed between species. By comparing changes observed in the physiological variables measured, we can better understand the unique physiological responses to both temperature and *p*CO_2_ found within each species.

With respect to elevated temperature, there were significant changes in cell volume and biochemical composition in *Symbiodinium C15* and *S*. *trenchii* (Fig. 3), whereas there were no changes for the more thermally sensitive symbionts *C21a* and *C1c-d-t*. In agreement, heat-induced reductions in PSII efficiency were more pronounced in symbionts *C21a* and *C1c-d-t* as compared to *C15* and *S. trenchii*, and are likely due to an initial increase in photo-stress within the photosynthetic apparatus. While the photochemical response of *S. trenchii* noted here is consistent with thermal tolerance, there was a significant reduction in F_v_/F_m_ under elevated temperature and ambient *p*CO_2_ in the *C15* symbiont. Additionally, reductions in Fv/Fm in the *C15* symbiont were also observed with increasing *p*CO_2_. This contrasts with Wall *et al.* (2013) where there was no change in maximal PSII efficiency in *Seriatopora caliendrum* due to elevated *p*CO_2_ exposure^33^. It is also likely that the photochemical responses noted here were influenced by relatively low light levels (275 μmol quanta m^−2^ s^−1^), as previous work has highlighted the importance of light intensity in the physiological response to elevated temperature^34^ and *p*CO_2_^35^.

The contrasting trends in PSII photosynthetic efficiency, along with changes in algal density and size may point to important differences in response to high temperature between thermally tolerant vs. sensitive symbionts. We have previously shown that there was a significant drop in *C15* density with elevated temperature (described in Schoepf *et al.* 2013) which may have increased the internal light field for the remaining symbionts^36^, resulting in a high-light acclimation phenotype. Previous studies have provided a clear link to internal light fields playing a substantial role to the bleaching response^37^ with high light intensity exacerbating thermal stress. It is interesting to note that increased cell volume is a common strategy for high light acclimation within many phytoplankton species^38^, and may also have played a similar function for the *C15* symbiont. Despite thermal reductions in algal density only occurring under high *p*CO_2_ in *T. reniformis*, an increase in cell volume could still play a similar role in changing light fields for *S. trenchii*. While not reported here, pigment analysis from this study did result in a slight yet significant thermal rise in chlorophyll *a* cell^−1^ in *T. reniformis* (Pettay *et al.* in prep). An increase in cell volume would help offset potential increases in optical absorption cross section due to increases in chlorophyll density^39^. In addition, although thermally-induced changes in biochemical composition were observed for *S. trenchii* and *C15*, they differed with respect to which component (i.e., proteins or lipids) changed significantly (Fig 4), likely also influencing the direction of thermal change between the two species as observed in Figure 1. This may indicate differences in the biochemical pathways that correlate to specific changes in light absorption and thermal tolerance. Indeed, recent large-scale metatranscriptome analyses of phytoplankton across different ocean provinces have noted the incredible influence that temperature has over metabolic variability^40^. While protein cell^−1^ often rises with temperature in other phytoplankton^41^, shifting lipid composition has also been noted^42^.

Thicker coral tissue could provide greater symbiont photoprotection by changing the intensity and spectral properties of the internal light field^43^,^44^ and higher levels of energy reserves (the sum total of lipid, protein and carbohydrate) have been implicated in facilitating bleaching resistance in some Caribbean corals^7^, Hawaiian^8^, and Australian^9^ corals. Notably, coral tissue biomass was roughly 25% higher in *T. reniformis* as compared to the other three species^11^. In addition, host protein concentrations in *T. reniformis* were almost twice as high *A. millepora* and *P. damicornis* (Fig 5). At the same time, carbohydrate concentration in both *M. monasteriata* and *T. reniformis* were roughly 5–10 fold greater than either *A. millepora* or *P. damicornis* (Fig 5). This higher biomass may have contributed to providing the symbionts with additional photoprotection and a significant advantage within a high temperature and/or high CO_2_ environment.

Interestingly, host protein, lipid and carbohydrate concentrations within this study differ from Schoepf *et al.*, (2013) where whole coral lipid concentrations for *A. millepora* declined at elevated temperature but only at the highest *p*CO_2_ level, whereas few physiological changes were observed within the other three species^11^. However, coral samples were sampled only from the growth tip of each coral species^11^. As skeletal porosity and thickness, along with symbiont cell density, photopigment concentrations, and lipid concentrations may increase with distance from the growth tip^45^,^46^,^47^, it is likely that the biochemical composition of the symbiosis does as well. Therefore our approach here was to integrate these metrics over the whole coral fragment and quantify host and symbiont biochemical composition separately. As a result, the different trends in host soluble protein and carbohydrates between this study and Schoepf *et al* (2013) likely reflect spatial differences in physiological function and biochemical makeup between the growing tip (or edge) and the rest of the coral colony as a whole. Such spatial differences may be important in differentiating effects on short-term colony growth versus long-term colony maintenance, and understanding if environmental stress differentially affects small coral recruits (which are likely most similar to coral tips) as compared to larger adult colonies.

The animal host fraction of *A. millepora* and *M. monasteriata* biochemical composition declined the most in response to elevated temperature (Fig 5), yet no thermally-induced differences were observed for *Symbiodinium C21a*, while *C15* in *M. monasteriata* increased soluble protein and lipid concentrations with temperature (Fig 4). Increased symbiont lipid production, along with reductions in host energetic reserves in *M. monasteriata*, likely imposed substantial metabolic demand on the holobiont and may have contributed to the greater LEDR at high temperature. In contrast, few physiological changes were observed for *P. damicornis*, yet equally large increases in LEDR were noted, suggesting different oxygen consuming pathways are responsible for increased respiration in each species. In addition to thermal enhancement of respiration, divergent use of oxygen consuming pathways within the symbiont may play a role. Photorespiration, alternative oxidase, and especially the Mehler ascorbate peroxidase cycle are all mechanisms possibly used for energy regulation in different phytoplankton, including *Symbiodinium*^48^,^49^. Likewise, several of these pathways have been implicated in different *Symbiodinium* subjected to high temperature^2^,^50^.

Previous studies have reported a significant drop in coral carbonic anhydrase (CA) expression during acute heat^51^,^52^ and combined high temperature and *p*CO_2_ exposure^53^. In contrast, we note a temperature-driven increase for intracellular CA in *A. millepora* (Fig. 5a). If the intracellular carbonic anhydrase in *A. millepora* is heavily localized within the gastrodermal tissue layer, as in the coral *Stylophora pistillata*^54^, thermal up-regulation of this CA isoform could indicate enhanced host delivery of carbon to the symbiont. *A. millepora* also displayed a significant increase in net photosynthesis under high temperature (Pettay at al. in prep, data not shown) indicating a potential link between enhanced carbon delivery by the host and symbiont productivity. However, thermal reductions in Fv/Fm were also noted for *A. millepora* and further complicate the issue. The lack of change in CA expression in *A. millepora* with respect to *p*CO_2_ concentration is consistent with previous OA experiments with adult *A. millepora* colonies^23^.

Because both extracellular CA and CA-ATPase genes are thought to be involved in calcification^27^, it is of interest that no change in expression was noted for *A. millepora* despite *p*CO_2_ induced reductions in calcification^11^. In contrast, CA expression drops significantly under elevated CO_2_ in *A. millepora* planulae larvae undergoing initial stages of settlement and skeletal formation^22^, showing that the discrepancies between studies may be driven in part by different life stages tested. The complete lack of changes in either of the carbonic anhydrase or CA-ATPase genes tested within *P. damicornis* may indicate that carbon concentrating mechanisms may differ among species.

The increase in transcripts for heat shock protein 90 (HSP90) in *A. millepora* under high temperature and medium *p*CO_2_ agrees with previous studies, which documented up-regulation of heat-shock proteins as an important component of the thermal stress response^55^,^56^. However, for *A. millepora*, this increase in HSP90 transcript was not observed in the highest or lowest *p*CO_2_ treatment and may indicate a sensitivity in the host thermal response to *p*CO_2_. Significant thermal up-regulation of HSP90 transcripts were observed for *P. damicornis*, with the most pronounced increase occurring within the low *p*CO_2_ treatments (Fig 5j), again suggesting that the thermally induced increase in HSP90 may change with *p*CO_2_. As some heat shock proteins play a fundamental role in protein stabilization, the low pH associated with OA may also affect protein conformation, further complicating the HSP90 response under elevated temperature and high CO_2_^57^. Lack of change in expression of GAPDH within either species is not surprising, as previous studies have suggested its relatively stable transcription rate can be utilized as a housekeeping gene for normalization of qPCR data^58^.

Studies of nuclear encoded gene expression in *Symbiodinium* typically note small to minimal changes, even when significant thermal and or light stress is applied^59^,^60^. This is likely a consequence of greater dependency on post-transcriptional regulation within dinoflagellates in general^61^,^62^. Nevertheless, our results demonstrate distinct expression patterns for two clade C symbionts in two different host species, thus illustrating the physiological diversity contained among different host/symbiont combinations. The symbiont genes studied here represent various metabolic pathways, including nitrogen metabolism (glutamine synthetase), the citric acid cycle (α-ketoglutarate dehydrogenase) and fatty acid synthesis (malonyl Co-A acyl transferase). Interestingly, high temperature-induced down-regulation of glutamine synthetase (*GSII*) in *Symbiodinium C1c-d-t* is similar to the reduction in *GSII* transcripts within the diatom *Thalassiosira pseudonana* and may indicate a reduction in nitrogen metabolism from nitrate^63^. Although minimal, the increase in α-ketoglutarate dehydrogenase gene expression with increasing *p*CO_2_ in *C21a* correlated with the decline in carbohydrates, possibly indicating an increase in the citric acid cycle, which links amino acid synthesis and breakdown with sugar metabolism. While expression of malonly Co-A acyl transferase in *Symbiodinium C21a* increased, this may not suggest enhanced lipid synthesis at high *p*CO_2,_ as there was little evidence for this based on cellular lipid content (Fig. 4i). However, it is possible that additional lipids synthesized by the symbiont were translocated to the host, where evidence of significant lipid catabolism in response to both temperature and *p*CO_2_ was noted.

Similarity analyses indicate that the physiological variables that explain the largest change in one species may be less important in explaining physiological change in another. For example, with respect to temperature, host parameters contributed the most to the physiological response of *A. millepora*, whereas symbiont metrics changed to a greater extent in *T. reniformis*. This highlights the uniqueness of each holobiont as host and symbiont tolerances to environmental stress may differ and greatly influence the resulting physiological responses. In addition, our results indicate that focusing on just a few key variables may not capture the full breadth of physiological change that may occur in response to thermal stress.

When directly comparing trends in each variable, substantial shifts in cell volume and biochemical composition within *Symbiodinium C15* and *S. trenchii* point to potential strategies in thermal tolerance and acclimation not observed within *C21a* and *C1c-d-t*. With respect to elevated *p*CO_2_, increased expression of α-ketoglutarate dehydrogenase correlated with declines in carbohydrate concentration for *C21a* and may reflect an increase in the citric acid cycle. In contrast, protein concentrations within the other two clade-C symbionts increased with *p*CO_2_ and reflect the physiological differences amongst symbiont types at the intra-cladal scale. Furthermore, differing thermal responses in biochemical composition within *A. millepora* and *M. monasteriata* belie similarities in respiration rates, and suggest that reliance on a few physiological metrics may not fully characterize nuanced physiological differences in response to environmental change. Our results suggest that conclusions based on experimental work may only be applicable to the host/symbiont combination in question and care should be taken in attempting to apply “species-specific” responses towards a more general understanding of coral reef system-wide effects from climate change. Overall, elevated *p*CO_2_ induced very little change across all species, as compared to elevated temperature. However, these results are based on a relatively short-term exposure to OA as compared to what future reefs will endure. Therefore, further studies are required to better understand the extent of physiological change under long-term elevated *p*CO_2_.

## Materials and Methods

### Experimental Design

A detailed description of the experimental design is found in^11^. Briefly, six colonies of *Acropora millepora, Pocillopora damicornis*, *Montipora monasteriata*, and *Turbinaria reniformis* were collected in northwest Fiji at a depth between 3–10 m, transported to a coral mariculture facility (Reef Systems Coral Farm, Ohio), and allowed to acclimate for two months prior to experimentation. Six fragments were removed from each parent colony species^−1^ and allowed to recover. Coral fragments were slowly acclimated (over a three weeks) to custom-made synthetic seawater closely resembling natural seawater chemistry (ESV Aquarium Products Inc.). There were 6 treatment systems, each consisting of six, 57 L aquaria connected to a central 905 L sump. One colony fragment species^−1^ was placed into each of the six replicate tanks in each system, with a separate colony fragment from each species in each replicate tank (i.e., four fragments per tank). Because replicate tanks were connected via a central sump per treatment, our design is technically a pseudo-replicate design^64^. However, controlling temperature, CO_2_, salinity and light intensity within individual replicate tanks is technically difficult and impractical for this type of study. Corals were maintained under a 9:15 hour light:dark cycle, providing light at 275 μmol quanta m^−2^ s^−1^ at the base of the filled aquaria. Each treatment ran for 24 days, with a 25% water change every three days. Salinity was maintained at 35 ppt through daily top-offs with RO filtered fresh water. Corals were fed *Artemia nauplii* every three days.

Treatments consisted of an ambient and high temperature treatment at three *p*CO_2_ conditions set to ambient (382 μatm), medium (607 μatm) and high CO_2_ (741 μatm). The *p*CO_2_ conditions reflect current (382 μatm) and elevated conditions expected by the mid (607 μatm) and late (741 μatm) 21^st^ century^65^. Temperature within the high temperature treatments was slowly increased with titanium heaters from the ambient temperature of 26.5 °C to a maximum of 31.5 °C over the course of the experiment (see supplemental Figure 1 for temperature ramping profiles).

pH measurements were taken every 30 seconds (Thermos Scientific Orion Ross Ultra pH glass electrode) and were integrated into a pH stat system for precise control of air and CO_2_ gas input into each sump (KSgrowstat, University of Essex). For elevated *p*CO_2_ treatments, CO_2_ was increased by 100 μatm day^−1^ until the desired *p*CO_2_ was met. All pH electrodes were calibrated daily using three NBS standards (4.00, 7.00, 10.00), and independent measurements of pH with a dedicated Ross pH electrode and alkalinity with Gran titration were made using published protocols^66^. Total alkalinity, pH, salinity, and temperature over the 24-day experiment were used to calculate carbonate system speciation using the CO2SYS program (Lewis & Wallace, 1998). The results are shown in supplementary table 1.

### Symbiont Photophysiology

Daily dark acclimated maximum quantum yield of photosystem II (F_v_/F_m_) was measured one hour after the light period by pulse amplitude modulation fluorometry (Diving PAM, Waltz, Germany), by supplying a 600 ms pulse of saturating light to the surface of each coral fragment. On day 23, maximal photosynthetic rates and light acclimated dark respiration (R_L_) were measured for 6 fragments species^−1^ treatment^−1^ via respirometry with galvanic oxygen electrodes (Qubit systems) housed in clear acrylic chambers (350 mL). Chambers were temperature controlled to match experimental conditions and constantly stirred. Maximal photosynthesis was measured by providing illumination from a 24 LED array (Cree Cool White XP-G R5) set to 600 μmol quanta m^−2^ s^−1^. Pilot experiments at this light intensity showed no decrease in oxygen evolution (data not shown). Net maximal photosynthesis (Pmax_net_) was recorded for 15–20 minutes, followed by a 10-minute dark incubation to record the light enhanced dark respiration (R_L_) also known as the post-illumination respiration (referred to hereafter as the LEDR). The photosynthesis to respiration ratio was calculated as Pmax_gross_/R_L_ where Pmax_gross_ = (net photosynthesis + light enhanced dark respiration). Net photosynthesis (data not shown) and light enhanced dark respiration (R_L_) were normalized to total fragment surface area (cm^2^) (described below).

### Host and Symbiont Physiology

At the end of the 24-day treatment, samples were frozen in liquid N_2_ and stored at −80 °C until further processing. All coral tissue was removed from each fragment using a water pick^67^. This is fundamentally different from our companion paper^11^ based on the same experiment, where only tissue from the branch tips or the leading edge of plating corals were analyzed (with the exception of cell density and chlorophyll content which were both integrated from the whole fragment). In contrast, this paper focuses on the physiological responses integrated over the entire fragment area. The resulting slurry was homogenized with a tissue tearer (Biospec products, Inc), and then centrifuged for 5 minutes (5,000 g) to separate the algal and coral fractions. Pelleted symbionts were resuspended in synthetic seawater and divided into 1 mL aliquots. One algal aliquot was preserved with 10 μL of 1% glutaraldehyde for cell enumeration, and cell density and volume were recorded by light and fluorescence microscopy. Six independent replicate counts were performed for each algal sample on a hemocytometer. Samples were photographed using a Nikon microphot-FXA epifluorescent microscope (100x magnification) and analyzed using the software Image J (NIH) with the Analyze Particles function. Pixel size of each cell was converted to μm^2^ using a calibrated scale micrometer and then used to calculate cell diameter and volume based on calculations for a sphere. Surface area of *T. reniformis* and *M. monasteriata* was determined by the foil method^68^, while area for the branching *A. millepora* and *P. damicornis* was determined by the wax method^69^.

For soluble protein concentration of the host and symbiont, 1 mL samples were homogenized with a bead-beater (BioSpec) for 2 minutes and then analyzed using the BCA protein method (Thermo Scientific Pierce), with a bovine serum albumin standard^70^. For lipid extraction, host and symbiont portions were freeze-dried overnight and then extracted using a 2:1:0.8 chloroform:methanol:sodium chloride ratio^71^. Lipid quantification was carried out by a vanillin colorimetric assay using corn oil as standards^72^. For carbohydrate quantification, host and symbiont aliquots were homogenized with a bead-beater for 1.5 minutes and then extracted using a sulfuric acid/phenol, using glucose as standard^73^. Absorbance measurements for lipid, carbohydrate and protein assays were made at 540, 485 and 595 nm respectively using a FLUOstar Omega plate reader (BMG Labtech, Germany). Biochemical composition was normalized to coral surface area and algal cell number.

The genetic identity of the dominant algal symbiont of each coral fragment was determined through amplification of the internal transcribed spacer 2 region (ITS2) of the ribosomal array, and subsequently analyzed by previously published protocols for denaturing gradient gel electrophoresis (DGGE) fingerprinting and cycle sequencing^74^. This method identifies the dominant (or co-dominant) sequence variants for the ribosomal genome of a particular symbiont lineage.

### Targeted Host and Symbiont Gene Expression

Due to the greater availability of genomic data for the coral *A. millepora* and *P. damicornis* and their respective *Symbiodinium*, gene expression was examined only in these two species and their respective symbionts. Transcript abundance for host genes involved in several metabolic roles was investigated, including carbon acquisition (intra and extracellular carbonic anhydrase), calcium and ATP exchange (CA-ATPase), carbon metabolism (Glyceraldehyde 3-phophate dehydrogenase (GAPDH)) and thermal response (Heat Shock Protein 90 (HSP90)). For algal gene expression, transcript abundance was monitored in pathways related to carbon metabolism (α-ketoglutarate dehydrogenase), nitrogen metabolism (glutamine synthetase), and fatty acid synthesis (malonyl Co-A acyl transferase)^52^.

For intra and extracellular carbonic anhydrases and Ca-ATPase*, A. millepora* and *P. damicornis* expressed sequence tags were searched using a conventional BLAST search within the National Center for Bioinformatics (NCBI) and PocilloporaBase (cnidarians.bu.edu/PocilloporaBase/cgi-bin/index.cgi) databases respectively. Databases were queried with well-characterized gene transcripts from *Stylophora pistillata*, intra and extracellular carbonic anhydrase (STPCA-2, EU532164.1 and STPCA EU159467.1 respectively) and CA-ATPase (AY360080.1)^27^,^54^,^75^. Databases were also searched using the sequences for GAPDH (EZ026309.1) and HSP90 (DY584045.1) from *Acropora aspera*^52^. The top blast hits were confirmed as homologous genes through phylogenetic analysis (Geneious, Biomaters Ltd). All coral genes were normalized to transcripts encoding ribosomal subunit protein 7 (rsp7) and Elongation factor 1 α (EF1*α*)^58^. Algal transcripts were normalized to the housekeeping genes encoding S-adenosyl methionine synthetase (SAM)^59^ and the proliferating cell nuclear antigen (PCNA)^52^. Primers for qPCR were designed with Primer-Quest software (Integrated DNA Technologies) or from published studies^52^,^58^. Primer sets and efficiency scores for each gene are listed in supplementary table 2.

Frozen coral samples were ground into a powder using a mortar and pestle chilled on a bed of dry ice. Total RNA was extracted and purified from each sample using TRIzol Reagent (Invitrogen) and an Aurum Total RNA Mini kit (Bio-Rad). Purified RNA samples were quantified by spectrophotometry (NanoDrop 2000, ThermoScientific). Only samples with concentrations greater than 50 ng μL^−1^ were used for subsequent analyses, and samples typically had 260 nm:280 nm and 260 nm:230 nm ratios greater than 2.0 and 1.9 respectively. RNA samples were diluted to 20 ng μL^−1^ prior to cDNA synthesis. Reverse transcription PCR reactions were performed using the high capacity cDNA Reverse Transcription Kit (Applied Biosystems). For each sample, a total of 120 ng of total RNA was added to a single 10 μl cDNA reaction.

Quantitative PCR reactions were performed on 96 well plates with optical film, using a SensiMix real time detection system with 2X SYBR Hi-ROX Mastermix (BIOLINE) and an ABI Prism 7500 Sequence Detection System (Applied Biosystems). Each 10 μL reaction contained 5 μL of SYBR Hi-ROX, 0.2 μL each of 10 mM forward and reverse primer, 2.6 μL nuclease-free water and 2 μL of 1:5 diluted cDNA. All qPCR reactions were performed with the following thermal profile: 50 °C for 2 min, 95 °C for 10 min, followed by 40 cycles of 95 °C for 15s, 61 °C for 15s and 72 °C for 45s. Standards were constructed from pooled total RNA samples from multiple treatments and diluted in a 4-fold dilutions series. For each plate, standards were run in triplicate and samples run in duplicate. A dissociation curve between 61 °C and 95 °C (0.5 °C intervals) was performed immediately after each PCR reaction to ensure the absence of any non-specific, multi-product amplification. Negative control reactions were carried out on a subset of samples and pooled standards (not shown).

Standards were constructed from pooled total RNA samples from multiple time points and diluted in a 4-fold dilutions series prior to cDNA synthesis. Standards were run in triplicate and samples run in duplicate. Efficiency values for each gene were calculated using the formula *E* = 10^(−1/slope)^ and are available in supplementary table S3. GeneEx expression software was used to normalize all data to total RNA and to the multiple house-keeping genes listed above and to account for differences in amplification efficiency. As the goal of this work was to compare gene expression across multiple variables and treatments rather than to a single control treatment, relative expression values for each gene of interest were calculated by dividing each value by the highest value within each gene assay^76^,^77^.

### Statistical Analysis

For each species, the overall importance of elevated temperature and *p*CO_2_, were analyzed for their significance in separating samples using an ANalyses Of SIMilarities (ANOSIM) with 9,999 permutations. If resulting correlation from the ANOSIM was above 0.2, a subsequent SIMPER analysis was used to determine which variables contributed the greatest towards dissimilarity between treatment factor levels (2 temperature and 3 *p*CO_2_ levels) within each species. Lastly, physiological variables across all four coral species were analyzed using non-metric multidimensional scaling (nMDS) on Euclidean distances after log(x + 1) transformation^78^.

In order to better understand possible nuanced physiological changes within each species, individual variables were also analyzed. For each species, individual variables were tested for homogeneity of variance and normality of distribution using the Levene and Shapiro-Wilks tests, respectively. If either test was significant (p < 0.05), the data was log transformed. A two-way analysis of variance (ANOVA) was utilized to test for significant main and interactive effects of *p*CO_2_ and temperature. As our focus was primarily on the main effects, and because only two temperatures were utilized, significant temperature effects were not followed up with post-hoc analyses. Significant differences for *p*CO_2_, were followed by Tukey post-hoc testing to distinguish between the three *p*CO_2_ treatments. Significant interactive effects were followed up by a pairwise comparison among all 6 treatments (Tukey post-hoc). Alternatively, if data failed to meet assumptions of normality even after log-transformation, a Kruskal-Wallis test with multiple comparisons was used. All statistical analyses were performed using R software with the ‘vegan’, ‘car’ and ‘pgirmess’ packages installed. Resulting output from SIMPER, ANOVA and Kruskal-Wallis tests are provided as supplemental information.

## Additional Information

**How to cite this article**: Hoadley, K. D. *et al.* Physiological response to elevated temperature and *p*CO_2_ varies across four Pacific coral species: Understanding the unique host+symbiont response. *Sci. Rep.* **5**, 18371; doi: 10.1038/srep18371 (2015).

## Supplementary Material

Supplementary Information

## Figures and Tables

**Table 1:** Contribution of temperature and CO_2_ to changes in 11 different variables within the four coral species, *A. millepora, P. damicornis, M. monasteriata, T. reniformis.*

	Source of variation	r	*p* value
*A. millepora*	Temperature	**0.2121**	**1.00E-04**
	CO_2_	**0.1218**	**0.0051**
*P. damicornis*	Temperature	**0.1787**	**4.00E-04**
	CO_2_	0.05743	0.1293
*M. monasteriata*	Temperature	**0.4117**	**1.00E-04**
	CO_2_	0.03002	0.2199
*T. reniformis*	Temperature	**0.3437**	**1.00E-04**
	CO_2_	**0.1034**	**0.0234**

ANalysis Of SIMilarity (ANOSIM with 9,999 permutations).
