# Supplementary material for: Physiological response to elevated temperature and pCO2 varies across four Pacific coral species: Understanding the unique host+symbiont response
Source: Sci Rep. 2015 Dec 16;5:18371. doi: 10.1038/srep18371 (PMC4680954; doi:10.1038/srep18371)
Supplement: Supplementary Information [file srep18371-s1.doc]

**Title:** Physiological response to elevated temperature and *p*CO_2_ varies across four Pacific coral species: Understanding the unique host+symbiont response.

**Author list**

Kenneth D. Hoadley^1,*^, D. Tye Pettay^1^, Andréa G. Grottoli^2^ , Wei-Jun Cai^3,✖^, Todd F. Melman^4^, Verena Schoepf^2^,**^☐^**, Xinping Hu^3,†^, Qian Li^3,‡^, Hui Xu^3,∆^, Yongchen Wang^3^, Yohei Matsui^2^, Justin H. Baumann^2^, Mark E. Warner^1^,

**Figure S1.** Thermal profiles for each experimental system. **LTLC:** Low Temperature, Low CO_2_; **HTLC:** High Temperature, Low pCO_2_; **LTMC:** Low Temperature, Medium pCO_2_; **HTMC:** High Temperature, Medium pCO_2_; **LTHC:** Low Temperature, High pCO_2_; **HTHC:** High Temperature, High pCO_2_.

**Table S1**. Mean (± 1 SE) carbonate chemistry parameters for 6 treatments representing three pCO_2_ levels at two temperatures. From Schoepf et al (2013).

|  | **Ambient pCO_2_** | | **Medium pCO_2_** | | **High pCO_2_** | |
| --- | --- | --- | --- | --- | --- | --- |
|  |  |  |  |  |  |  |
| **Temp. (°C)** | Ambient | Elevated | Ambient | Elevated | Ambient | Elevated |
| **pH_T_** | 8.07 ± 0.009 | 8.04 ± 0.013 | 7.90 ± 0.011 | 7.89 ± 0.010 | 7.83 ± 0.012 | 7.81 ± 0.014 |
| ***p*CO_2_**  **(μatm)** | 364.31± 9.69 | 400.62 ± 16.83 | 598.37 ± 18.50 | 616.08 ± 24.24 | 732.04 ± 22.37 | 749.63 ± 26.21 |
| **TA**  **(µmol kg^-1^)** | 2269.4 ± 10.84 | 2270.1 ± 11.15 | 2303.8 ± 9.34 | 2288.3 ± 10.43 | 2306.3 ± 10.64 | 2304.5 ± 9.08 |
| **Ω_arag_** | 3.69 ±  0.07 | 3.79 ±  0.09 | 2.75 ±  0.05 | 2.91 ±  0.05 | 2.40 ±  0.06 | 2.52 ±  0.06 |

**Table S2.** Quantitative real-time PCR primer sets and efficiencies. All *P. damicornis* contig ID numbers are from PocilloporaBase (Cnidarians.Bu.edu).

| **Gene ID** | **Forward (5’-3’)** | **Reverse (5’-3’)** | **Efficiency** | **Accession number, citation or contig ID number** |
| --- | --- | --- | --- | --- |
| ***A. millepora*** | | | | |
| *GAPDH* | ACCATCCATGCTTACACTGCGACA | AGGAATCACCTTTCCCACAGCCTT | 100.5 | EZ026309.1 |
| *HSP90* | ACTCCGCGAGCAACCATAAACTCT | AGCCTGCGCTCTTTCTTTGACTCT | 104.9 | EZ012996 |
| *Ca-ATPase* | TTGCTCCAGAAGAGTGCGAAGGTT | TACTTTGCAGGCCACGAAACTGCT | 102.3 | EZ002337.1 |
| *CA-IN* | GGCAAAGAAATACAAGTTCGAGC | TGTGTCTCGCAATCCCAATG | 106.2 | EU863783.1 |
| *CA-EX* | TCGGTGAAGATTGGAGTTACAG | AGTTGGTCAAGGTGAAGCTC | 103.5 | EU863782.1 |
| *RP-s7* | AGCAAAGGAGGTTGATGTGG | GACGGGTCTGGATCTTTTGA | 107.7 | Seneca 2010 |
| *EF1-a* | TGGCTTTTGTACCTATCTCTGG | TTGTCCAGTGCGTCGATAAG | 107.7 | GO003400 |
| ***P. damicornis*** | | | | |
| *GAPDH* | TTGGAAGACCGGCAGCCTTGTTAT | ACAGATTTGCCTGGCGCTAACACT | 101.3 | bu_91849.1_c2778 |
| *HSP90* | AAACATGCCCTGATGGAGCGAGTT | TAGTGGCTGGTGGACTGGCATCTAAT | 97.6 | bu_91849.1_c7242 |
| *Ca-ATPase* | TCAAGGGCATCATTGACAGCAAGC | TCTGTTCCAGCAATACCCATGGCA | 103 | bu_91849.1_c48148 |
| *CA-IN* | ACTGCACGCTCATGTAAGGGACAA | CTCTGGCTGAATGCGTCAAATGGA | 98.4 | bu_91849.1_c16633 |
| *CA-EX* | ACATCAGAGCGACGCGGAAAGTTA | ACCAGTTGCTGGTGCAGGCATTAT | 96.8 | bu_91849.1_lrc17419 |
| *RP-s7* | TCAGGAACAGGCGAGCCAAATGAA | AAGCTGCGACTTGAGATCGGTAGA | 99.6 | bu_91849.1_c20236 |
| *EF1-a* | TTGGTTGTTGCTGCTGGTACTGGT | ATACTTGGGCTCAGTGGTGTCCAT | 99.6 | AB245432.2 |
| ***Symbiodinium*** | | | | |
| *GLUS* | GAGGAAACCGAGGCCCGTCAGGAAGTGA | AGCAGGCAAGAAGCCGGTCTCGGTCATC | 103 | Leggat 2011 |
| *KETO* | ATCAAGATGCGAAAGGAATACAAG | GCATAAGCACTGGCCAGAAAGAAC | 101.3-105 | Leggat 2011 |
| *McOA* | TTTCCAAAGGGCTTCTCGTGTGC | ACCCTTCTTCTCAGCCAGCTCCTTCAG | 101.3-102 | Leggat 2011 |
| *SAM* | GCCTACATTTGCCGACAGATG | AATGGCTTGGCAACACCAAT | 94.6-102 | Leggat 2011 |
| *PCNA* | GAGTTTCAGAAGATTTGCCGAGAT | ACATTGCCACTGCCGAGGTC | 99.6-102 | Leggat 2011 |

**Statistical tables for all univariate analysis.** **LTLC:** low temperature, low CO_2_; **HTLC:** high temperature, low CO_2_; **LTMC:** low temperature, medium CO_2_; **HTMC:** high temperature, medium CO_2_; **LTHC:** low temperature, high CO_2_; **HTHC:** high temperature, high CO_2_; **HT:** high temperature treatments**; LT:** low temperature treatments; **LC:** low CO_2_ treatments; **MC:** medium CO_2_ treatments; **LC:** low CO_2_ treatments. **Temp = temperature**

**Supplemental Table 3: *Photosynthetic Efficiency of PSII***

| **Kruskal Wallace** | **Effect** | **p-value** | **pair-wise** | **HTHC** | **HTLC** | **HTMC** | **LTHC** | **LTLC** |
| --- | --- | --- | --- | --- | --- | --- | --- | --- |
| ***C21a (A. millepora)*** | Temperature | **8.20E-06** | HTLC | 0.8719 | - | - | - | - |
| **Fv/Fm** | CO2 | non sig | HTMC | 0.8066 | 1 | - | - | - |
|  |  |  | LTHC | **0.0093** | 0.2105 | 0.275 | - | - |
|  |  |  | LTLC | **0.0148** | 0.275 | 0.35 | 1 | - |
|  |  |  | LTMC | **0.0067** | 0.1719 | 0.2287 | 1 | 0.9999 |
|  |  |  |  |  |  |  |  |  |
| **Kruskal Wallace** | **Effect** | **p-value** | **pair-wise** | HTHC | HTLC | HTMC | LTHC | LTLC |
| ***C1c-d-t (P. damicornis)*** | Temperature | **3.50E-07** | HTLC | 0.9989 | - | - | - | - |
| **Fv/Fm** | CO2 | non sig | HTMC | 1 | 0.9973 | - | - | - |
|  |  |  | LTHC | **0.0129** | **0.0033** | **0.017** | - | - |
|  |  |  | LTLC | 0.0754 | **0.0251** | 0.0932 | 0.9918 | - |
|  |  |  | LTMC | 0.1142 | **0.0411** | 0.1386 | 0.975 | 1 |
|  |  |  |  |  |  |  |  |  |
| **Two-Way ANOVA** | **Effect** | **Df** | **SS** | **Mean Sq** | **F-statistic** | **p-value** |  |  |
| ***C15 (M. monasteriata)*** | Temperature | 1 | 0.013754086 | 0.013754086 | 14.813643 | 0.000577484 |  |  |
| **Fv/Fm** | CO2 | 2 | 0.001033265 | 0.000516632 | 0.5564315 | 0.579054114 |  |  |
|  | Temp*CO2 | 2 | 0.013465859 | 0.00673293 | 7.2516061 | **0.002697419** |  |  |
|  |  |  |  |  |  |  |  |  |
| **Two-Way ANOVA** | **Effect** | **Df** | **SS** | **Mean Sq** | **F-statistic** | **p-value** |  |  |
| ***S. trenchii*** | Temperature | 1 | 0.000233411 | 0.000233411 | 0.1963175 | 0.6608886 |  |  |
| **Fv/Fm** | CO2 | 2 | 0.005149653 | 0.002574826 | 2.1656348 | 0.1322729 |  |  |
|  | Temp*CO2 | 2 | 0.001597135 | 0.000798567 | 0.6716589 | 0.5183753 |  |  |

**Supplemental Table 4: *Photosynthesis:Respiration and Light Enhanced Dark Respiration***

| **Two-Way ANOVA** | **Effect** | **Df** | **SS** | **Mean Sq** | **F-statistic** | **p-value** |
| --- | --- | --- | --- | --- | --- | --- |
| ***A. millepora*** | Temperature | 1 | 0.01161436 | 0.01161436 | 0.08406219 | 0.7740025 |
| **PR** | CO2 | 2 | 0.11041134 | 0.05520567 | 0.39956642 | 0.6743738 |
|  | Temp*CO2 | 2 | 0.06470174 | 0.03235087 | 0.23414843 | 0.7927781 |
|  |  |  |  |  |  |  |
| **Two-Way ANOVA** | **Effect** | **Df** | **SS** | **Mean Sq** | **F-statistic** | **p-value** |
| ***A. millepora*** | Temperature | 1 | 0.001611096 | 0.001611096 | 4.48637 | 0.04317498 |
| **LEDR** | CO2 | 2 | 0.002544945 | 0.001272472 | 3.543415 | 0.04248097 |
|  | Temp*CO2 | 2 | 0.002637175 | 0.001318588 | 3.671831 | **0.03835754** |
|  |  |  |  |  |  |  |
| **Two-Way ANOVA** | **Effect** | **Df** | **SS** | **Mean Sq** | **F-statistic** | **p-value** |
| ***P. damicornis*** | Temperature | 1 | 0.03119582 | 0.031195823 | 1.7619512 | 0.1943948 |
| **PR** | CO2 | 2 | 0.05016106 | 0.025080532 | 1.4165574 | 0.2583078 |
|  | Temp*CO2 | 2 | 0.01022753 | 0.005113764 | 0.2888272 | 0.7512012 |
|  |  |  |  |  |  |  |
| **Two-Way ANOVA** | **Effect** | **Df** | **SS** | **Mean Sq** | **F-statistic** | **p-value** |
| ***P. damicornis*** | Temperature | 1 | 0.049345424 | 0.049345424 | 23.766759 | 3.31E-05 |
| **LEDR** | CO2 | 2 | 0.007069672 | 0.003534836 | 1.702521 | 1.99E-01 |
|  | Temp*CO2 | 2 | 0.018384301 | 0.009192151 | 4.427313 | **2.07E-02** |

**Supplemental Table 4: Continued**

| **Two-Way ANOVA** | **Effect** | **Df** | **SS** | **Mean Sq** | **F-statistic** | **p-value** |
| --- | --- | --- | --- | --- | --- | --- |
| ***M. monasteriata*** | Temperature | 1 | 1.600893 | 1.600893 | 4.133195 | 0.05098114 |
| **PR** | CO2 | 2 | 0.3945851 | 0.1972926 | 0.509371 | 0.60597695 |
|  | Temp*CO2 | 2 | 1.9949465 | 0.9974733 | 2.575282 | 0.09286121 |
|  |  |  |  |  |  |  |
| **Two-Way ANOVA** | **Effect** | **Df** | **SS** | **Mean Sq** | **F-statistic** | **p-value** |
| ***M. monasteriata*** | Temperature | 1 | 0.074260104 | 0.074260104 | 21.3928375 | **6.70E-05** |
| **LEDR** | CO2 | 2 | 0.003188824 | 0.001594412 | 0.4593179 | 6.36E-01 |
|  | Temp*CO2 | 2 | 0.002684055 | 0.001342028 | 0.3866111 | 6.83E-01 |
|  |  |  |  |  |  |  |
| **Two-Way ANOVA** | **Effect** | **Df** | **SS** | **Mean Sq** | **F-statistic** | **p-value** |
| ***T. reniformis*** | Temperature | 1 | 0.7713854 | 0.7713854 | 0.5261016 | 0.4738708 |
| **PR** | CO2 | 2 | 0.4065798 | 0.2032899 | 0.1386481 | 0.8710889 |
|  | Temp*CO2 | 2 | 0.9256682 | 0.4628341 | 0.3156629 | 0.7316981 |
|  |  |  |  |  |  |  |
| **Two-Way ANOVA** | **Effect** | **Df** | **SS** | **Mean Sq** | **F-statistic** | **p-value** |
| ***T. reniformis*** | Temperature | 1 | 0.022615564 | 0.022615564 | 9.2055349 | **0.004946435** |
| **LEDR** | CO2 | 2 | 0.001244397 | 0.000622198 | 0.2532623 | 0.777907262 |
|  | Temp*CO2 | 2 | 0.003013143 | 0.001506571 | 0.6132412 | 0.548242709 |
|  |  |  |  |  |  |  |

***Supplemental Table 5:*** Symbiont protein, carbohydrate, lipid and cellular volume

| **Two-Way ANOVA** | **Effect** | **Df** | **SS** | **Mean Sq** | **F-statistic** | **p-value** | **Tukey** |
| --- | --- | --- | --- | --- | --- | --- | --- |
| ***C21a (A. millepora)*** | Temperature | 1 | 0.06425421 | 0.06425421 | 0.8275925 | 0.37022096 | LC=HC  MC>HC  MC=LC |
| **Symbiont Protein** | CO2 | 2 | 0.6209829 | 0.31049145 | 3.9991213 | **0.02886464** |
|  | Temp*CO2 | 2 | 0.23093761 | 0.1154688 | 1.487235 | 0.24218735 |
|  |  |  |  |  |  |  |  |
| **Two-Way ANOVA** | **Effect** | **Df** | **SS** | **Mean Sq** | **F-statistic** | **p-value** |  |
| ***C21a (A. millepora)*** | Temperature | 1 | 8.89E-15 | 8.89E-15 | 0.02420762 | 0.877400001 |  |
| **Symbiont Carboh** | CO2 | 2 | 5.18E-12 | 2.59E-12 | 7.0509905 | 0.003089921 |  |
|  | Temp*CO2 | 2 | 4.82E-12 | 2.41E-12 | 6.56843629 | **0.004306148** |  |
|  |  |  |  |  |  |  |  |
| **Two-Way ANOVA** | **Effect** | **Df** | **SS** | **Mean Sq** | **F-statistic** | **p-value** |  |
| ***C21a (A. millepora)*** | Temperature | 1 | 2.95E-10 | 2.95E-10 | 0.01201786 | 0.91343603 |  |
| **Symbiont Lipid** | CO2 | 2 | 1.59E-07 | 7.93E-08 | 3.2295768 | 0.05366858 |  |
|  | Temp*CO2 | 2 | 2.77E-08 | 1.38E-08 | 0.56331289 | 0.57522553 |  |
|  |  |  |  |  |  |  |  |
| **Two-Way ANOVA** | **Effect** | **Df** | **SS** | **Mean Sq** | **F-statistic** | **p-value** | **Tukey** |
| ***C21a (A. millepora)*** | Temperature | 1 | 4626.516 | 4626.516 | 0.7112414 | 4.06E-01 | LC=HC  LC<MC  MC>HC |
| **Cell Volume** | CO2 | 2 | 277391.555 | 138695.778 | 21.3219129 | **1.73E-06** |
|  | Temp*CO2 | 2 | 9814.963 | 4907.481 | 0.7544346 | 4.79E-01 |
|  |  |  |  |  |  |  |  |
| **Two-Way ANOVA** | **Effect** | **Df** | **SS** | **Mean Sq** | **F-statistic** | **p-value** |  |
| ***C1c-d-t (P. damicornis)*** | Temperature | 1 | 3.03E-06 | 3.03E-06 | 0.4242534 | 0.51995086 |  |
| **Symbiont Protein** | CO2 | 2 | 7.14E-05 | 3.57E-05 | 4.9993833 | 0.01363118 |  |
|  | Temp*CO2 | 2 | 6.39E-05 | 3.19E-05 | 4.4735648 | **0.02026173** |  |

**Supplemental Table 5: Continued**

| **Two-Way ANOVA** | **Effect** | **Df** | **SS** | **Mean Sq** | **F-statistic** | **p-value** |  |
| --- | --- | --- | --- | --- | --- | --- | --- |
| ***C1c-d-t (P. damicornis)*** | Temperature | 1 | 6.16E-12 | 6.16E-12 | 2.1261698 | 0.1551926 |  |
| **Symbiont Carboh** | CO2 | 2 | 2.25E-12 | 1.12E-12 | 0.3876247 | 0.6820169 |  |
|  | Temp*CO2 | 2 | 1.05E-11 | 5.23E-12 | 1.8031908 | 0.1821766 |  |
|  |  |  |  |  |  |  |  |
| **Two-Way ANOVA** | **Effect** | **Df** | **SS** | **Mean Sq** | **F-statistic** | **p-value** |  |
| ***C1c-d-t (P. damicornis)*** | Temperature | 1 | 1.42E-04 | 0.000141877 | 0.000368309 | 0.9848296 |  |
| **Symbiont Lipid** | CO2 | 2 | 3.76E-01 | 0.187816429 | 0.48756739 | 0.6194215 |  |
|  | Temp*CO2 | 2 | 9.36E-01 | 0.468015399 | 1.214957865 | 0.3124346 |  |
|  |  |  |  |  |  |  |  |
| **Two-Way ANOVA** | **Effect** | **Df** | **SS** | **Mean Sq** | **F-statistic** | **p-value** |  |
| ***C1c-d-t (P. damicornis)*** | Temperature | 1 | 7.54E+00 | 7.535757 | 0.00055118 | 0.9814251 |  |
| **Cell Volume** | CO2 | 2 | 3.87E+04 | 19362.45907 | 1.416207927 | 0.2583903 |  |
|  | Temp*CO2 | 2 | 4.45E+04 | 22260.8887 | 1.628204708 | 0.2131515 |  |
|  |  |  |  |  |  |  |  |
| **Two-Way ANOVA** | **Effect** | **Df** | **SS** | **Mean Sq** | **F-statistic** | **p-value** | **Tukey** |
| ***C15 (M. monasteriata)*** | Temperature | 1 | 0.52382161 | 0.52382161 | 7.2954821 | **0.01125692** | LC<MC  MC=HC  LC=HC |
| **Symbiont Protein** | CO2 | 2 | 0.53504901 | 0.2675245 | 3.7259254 | **0.03586902** |
|  | Temp*CO2 | 2 | 0.04103308 | 0.02051654 | 0.2857424 | 0.75347846 |
|  |  |  |  |  |  |  |  |
| **Two-Way ANOVA** | **Effect** | **Df** | **SS** | **Mean Sq** | **F-statistic** | **p-value** |  |
| ***C15 (M. monasteriata)*** | Temperature | 1 | 1.83E-07 | 1.83E-07 | 0.8703756 | 0.35829966 |  |
| **Symbiont Carboh** | CO2 | 2 | 1.08E-06 | 5.38E-07 | 2.5608217 | 0.09401484 |  |
|  | Temp*CO2 | 2 | 3.83E-07 | 1.92E-07 | 0.9121471 | 0.41251251 |  |

**Supplemental Table 5: Continued**

| **Two-Way ANOVA** | **Effect** | **Df** | **SS** | **Mean Sq** | **F-statistic** | **p-value** |  |
| --- | --- | --- | --- | --- | --- | --- | --- |
| ***C15 (M. monasteriata)*** | Temperature | 1 | 9.24E-07 | 9.24E-07 | 26.471077 | 1.70E-05 |  |
| **Symbiont Lipid** | CO2 | 2 | 1.10E-07 | 5.52E-08 | 1.581878 | 2.23E-01 |  |
|  | Temp*CO2 | 2 | 2.38E-07 | 1.19E-07 | 3.403997 | **4.70E-02** |  |
|  |  |  |  |  |  |  |  |
| **Two-Way ANOVA** | **Effect** | **Df** | **SS** | **Mean Sq** | **F-statistic** | **p-value** |  |
| ***C15 (M. monasteriata)*** | Temperature | 1 | 83087.23 | 83087.23 | 5.325177 | **0.02809773** |  |
| **Cell Volume** | CO2 | 2 | 49103.64 | 24551.82 | 1.573561 | 0.22393991 |  |
|  | Temp*CO2 | 2 | 49569.74 | 24784.87 | 1.588497 | 0.22093434 |  |
|  |  |  |  |  |  |  |  |
| **Two-Way ANOVA** | **Effect** | **Df** | **SS** | **Mean Sq** | **F-statistic** | **p-value** |  |
| ***S. trenchi*** | Temperature | 1 | 8.93483601 | 8.93483601 | 14.16275096 | **0.00072852** |  |
| **Symbiont Protein** | CO2 | 2 | 1.31446666 | 0.65723333 | 1.04179103 | 0.365238093 |  |
|  | Temp*CO2 | 2 | 0.04482602 | 0.02241301 | 0.03552722 | 0.965137008 |  |
|  |  |  |  |  |  |  |  |
| **Two-Way ANOVA** | **Effect** | **Df** | **SS** | **Mean Sq** | **F-statistic** | **p-value** |  |
| ***S. trenchi*** | Temperature | 1 | 1.37E-06 | 1.37E-06 | 5.1088584 | **0.03122135** |  |
| **Symbiont Carboh** | CO2 | 2 | 5.04E-07 | 2.52E-07 | 0.9407707 | 0.40154033 |  |
|  | Temp*CO2 | 2 | 7.68E-07 | 3.84E-07 | 1.4324963 | 0.25457499 |  |
|  |  |  |  |  |  |  |  |
| **Two-Way ANOVA** | **Effect** | **Df** | **SS** | **Mean Sq** | **F-statistic** | **p-value** | **Tukey** |
| ***S. trenchi*** | Temperature | 1 | 6.62E-13 | 6.62E-13 | 17.539846 | **0.000253298** | LC=MC  MC>HC  LC=HC |
| **Symbiont Lipid** | CO2 | 2 | 5.02E-13 | 2.51E-13 | 6.649796 | **0.004334828** |
|  | Temp*CO2 | 2 | 2.03E-13 | 1.02E-13 | 2.68976 | 0.085406567 |
|  |  |  |  |  |  |  |  |
| **Two-Way ANOVA** | **Effect** | **Df** | **SS** | **Mean Sq** | **F-statistic** | **p-value** | **Tukey** |
| ***S. trenchi*** | Temperature | 1 | 168144.174 | 168144.174 | 19.9607248 | **0.000104195** | LC>MC |
| **Cell Volume** | CO2 | 2 | 211103.567 | 105551.784 | 12.5302594 | **0.000110717** | MC<HC |
|  | Temp*CO2 | 2 | 4151.868 | 2075.934 | 0.2464382 | 0.783146329 | LC=HC |

**Supplemental Table 6: Figure 5-**Host protein, carbohydrate and lipids

| **Two-Way ANOVA** | **Effect** | **Df** | **SS** | **Mean Sq** | **F-statistic** | **p-value** | **Tukey** |  |  |
| --- | --- | --- | --- | --- | --- | --- | --- | --- | --- |
| ***A. millepora*** | Temperature | 1 | 1.0779601 | 1.0779601 | 12.0691802 | **0.001631836** | LC>MC  MC<HC  LC=HC |  |  |
| **Host Protein** | CO2 | 2 | 1.2015406 | 0.6007703 | 6.7264131 | **0.003982219** |  |  |
|  | Temp*CO2 | 2 | 0.02919521 | 0.01459761 | 0.1634394 | 0.84999472 |  |  |
|  |  |  |  |  |  |  |  |  |  |
| **Kruskal Wallace** | **Effect** | **Df** | **p-value** | **Pair-wise** | HTHC | HTLC | HTMC | LTHC | LTLC |
| ***A. millepora*** | Temperature | non sig | 0.3569 | HTLC | 1 | - | - | - | - |
| **Host Carboh** | CO2 | non sig |  | HTMC | 0.9 | 0.96 | - | - | - |
|  |  |  |  | LTHC | 0.95 | 0.89 | 0.39 | - | - |
|  |  |  |  | LTLC | 0.94 | 0.87 | 0.39 | 1 | - |
|  |  |  |  | LTMC | 1 | 1 | 0.97 | 0.86 | 0.84 |
|  |  |  |  |  |  |  |  |  |  |
| **Two-Way ANOVA** | **Effect** | **Df** | **SS** | **Mean Sq** | **F-statistic** | **p-value** | **Tukey** |  |  |
| ***A. millepora*** | Temperature | 1 | 246.83927 | 246.839269 | 17.4095884 | **0.000249988** | LC=MC  MC<HC  LC<HC |  |  |
| **Host Lipid** | CO2 | 2 | 215.8811 | 107.940551 | 7.6130535 | **0.002200035** |  |  |
|  | Temp*CO2 | 2 | 19.47047 | 9.735236 | 0.6866268 | 0.511265967 |  |  |
|  |  |  |  |  |  |  |  |  |  |
| **Two-Way ANOVA** | **Effect** | **Df** | **SS** | **Mean Sq** | **F-statistic** | **p-value** | **Tukey** |  |  |
| ***P. damicornis*** | Temperature | 1 | 50.23762 | 50.23762 | 0.005146079 | 0.943287983 | LC>MC  MC=HC  LC>HC |  |  |
| **Host Protein** | CO2 | 2 | 109790.3875 | 54895.19377 | 5.623176885 | **0.008434035** |  |  |
|  | Temp*CO2 | 2 | 59415.23826 | 29707.61913 | 3.043093315 | 0.062618687 |  |  |
|  |  |  |  |  |  |  |  |  |  |
| **Two-Way ANOVA** | **Effect** | **Df** | **SS** | **Mean Sq** | **F-statistic** | **p-value** | **Tukey** |  |  |
| ***P. damicornis*** | Temperature | 1 | 0.4076846 | 0.4076846 | 3.566584 | 0.06865682 | LC=MC  MC=HC  LC>HC |  |  |
| **Host Carboh** | CO2 | 2 | 1.003146 | 0.501573 | 4.387957 | **0.02130042** |  |  |
|  | Temp*CO2 | 2 | 0.5341872 | 0.2670936 | 2.336639 | 0.11399745 |  |  |

**Supplemental Table 6: Continued**

| **Two-Way ANOVA** | **Effect** | **Df** | **SS** | **Mean Sq** | **F-statistic** | **p-value** |  |
| --- | --- | --- | --- | --- | --- | --- | --- |
| ***P. damicornis*** | Temperature | 1 | 2152.1836 | 2152.184 | 0.2179618 | 0.6442098 |  |
| **Host Lipid** | CO2 | 2 | 977.2959 | 488.648 | 0.04948769 | 0.9517999 |  |
|  | Temp*CO2 | 2 | 4214.602 | 2107.301 | 0.21341633 | 0.8091216 |  |
|  |  |  |  |  |  |  |  |
| **Two-Way ANOVA** | **Effect** | **Df** | **SS** | **Mean Sq** | **F-statistic** | **p-value** |  |
| ***M. monsateriata*** | Temperature | 1 | 463267.11 | 463267.11 | 7.0900639 | **0.01234073** |  |
| **Host Protein** | CO2 | 2 | 21328.58 | 10664.29 | 0.1632115 | 0.85016068 |  |
|  | Temp*CO2 | 2 | 59633.23 | 29816.61 | 0.4563279 | 0.63793152 |  |
|  |  |  |  |  |  |  |  |
| **Two-Way ANOVA** | **Effect** | **Df** | **SS** | **Mean Sq** | **F-statistic** | **p-value** |  |
| ***M. monsateriata*** | Temperature | 1 | 2.088318 | 2.088318 | 9.0806056 | **0.005211103** |  |
| **Host Carboh** | CO2 | 2 | 0.900713 | 0.4503565 | 1.9582792 | 0.15872384 |  |
|  | Temp*CO2 | 2 | 0.1154596 | 0.0577298 | 0.2510257 | 0.779620208 |  |
|  |  |  |  |  |  |  |  |
| **Two-Way ANOVA** | **Effect** | **Df** | **SS** | **Mean Sq** | **F-statistic** | **p-value** |  |
| ***M. monsateriata*** | Temperature | 1 | 262.1559 | 262.15585 | 11.606965 | 0.002006491 |  |
| **Host Lipid** | CO2 | 2 | 168.2153 | 84.10767 | 3.723872 | 0.036810539 |  |
|  | Temp*CO2 | 2 | 272.1934 | 136.09669 | 6.025688 | **0.00666144** |  |
|  |  |  |  |  |  |  |  |
| **Two-Way ANOVA** | **Effect** | **Df** | **SS** | **Mean Sq** | **F-statistic** | **p-value** |  |
| ***T. reniformis*** | Temperature | 1 | 0.5877775 | 0.58777752 | 5.2269059 | **0.02947197** |  |
| **Host Protein** | CO2 | 2 | 0.0568307 | 0.02841535 | 0.2526881 | 0.77834665 |  |
|  | Temp*CO2 | 2 | 0.6450141 | 0.32250704 | 2.8679456 | 0.07248524 |  |
|  |  |  |  |  |  |  |  |
| **Two-Way ANOVA** | **Effect** | **Df** | **SS** | **Mean Sq** | **F-statistic** | **p-value** |  |
| ***T. reniformis*** | Temperature | 1 | 0.6396687 | 0.6396687 | 1.371227 | 0.25081861 |  |
| **Host Carboh** | CO2 | 2 | 1.1589829 | 0.5794914 | 1.242228 | 0.30316905 |  |
|  | Temp*CO2 | 2 | 4.4289894 | 2.2144947 | 4.747106 | **0.01617399** |  |

**Supplemental Table 6: Continued**

| **Two-Way ANOVA** | **Effect** | **Df** | **SS** | **Mean Sq** | **F-statistic** | **p-value** | **Tukey** |
| --- | --- | --- | --- | --- | --- | --- | --- |
| ***T. reniformis*** | Temperature | 1 | 0.08404074 | 0.08404074 | 0.08418799 | 0.77383825 | LC=MC |
| **Host Lipid** | CO2 | 2 | 9.43285477 | 4.71642738 | 4.72469141 | **0.01706032** | MC<HC |
|  | Temp*CO2 | 2 | 0.0263496 | 0.0131748 | 0.01319788 | 0.98689496 | LC<HC |

**Supplemental Table 7: Figure 6-**Host Gene Expression

| **Two-Way ANOVA** | **Effect** | **Df** | **SS** | **Mean Sq** | **F-statistic** | **p-value** |
| --- | --- | --- | --- | --- | --- | --- |
| ***A. millepora*** | Temperature | 1 | 0.72112297 | 0.72112297 | 48.642954 | **1.15E-07** |
| **Intercellular CA** | CO2 | 2 | 0.02881448 | 0.014407242 | 0.9718326 | 3.90E-01 |
|  | Temp*CO2 | 2 | 0.01338252 | 0.006691261 | 0.4513554 | 6.41E-01 |
|  |  |  |  |  |  |  |
| **Two-Way ANOVA** | **Effect** | **Df** | **SS** | **Mean Sq** | **F-statistic** | **p-value** |
| ***A. millepora*** | Temperature | 1 | 0.01131596 | 0.01131596 | 0.01436702 | 0.9054186 |
| **Extracellular CA** | CO2 | 2 | 1.02830797 | 0.51415398 | 0.65278234 | 0.528076 |
|  | Temp*CO2 | 2 | 0.20685355 | 0.10342678 | 0.13131314 | 0.8774616 |
|  |  |  |  |  |  |  |
| **Two-Way ANOVA** | **Effect** | **Df** | **SS** | **Mean Sq** | **F-statistic** | **p-value** |
| ***A. millepora*** | Temperature | 1 | 0.006332565 | 0.006332565 | 0.5978577 | 0.44565374 |
| **CA ATPase** | CO2 | 2 | 0.053055872 | 0.026527936 | 2.5045034 | 0.09923359 |
|  | Temp*CO2 | 2 | 0.01844037 | 0.009220185 | 0.870478 | 0.42940699 |
|  |  |  |  |  |  |  |
| **Two-Way ANOVA** | **Effect** | **Df** | **SS** | **Mean Sq** | **F-statistic** | **p-value** |
| ***A. millepora*** | Temperature | 1 | 0.03153086 | 0.03153086 | 2.599704 | 0.11809982 |
| **HSP90** | CO2 | 2 | 0.04527893 | 0.02263947 | 1.866613 | 0.17338571 |
|  | Temp*CO2 | 2 | 0.08226181 | 0.04113091 | 3.391223 | **0.04799221** |
|  |  |  |  |  |  |  |
| **Two-Way ANOVA** | **Effect** | **Df** | **SS** | **Mean Sq** | **F-statistic** | **p-value** |
| ***A. millepora*** | Temperature | 1 | 0.06001288 | 0.06001288 | 1.414271 | 0.24399508 |
| **GAPDH** | CO2 | 2 | 0.25454003 | 0.12727002 | 2.999262 | 0.06547115 |
|  | Temp*CO2 | 2 | 0.35853608 | 0.17926804 | 4.224654 | **0.02453782** |
|  |  |  |  |  |  |  |
| **Two-Way ANOVA** | **Effect** | **Df** | **SS** | **Mean Sq** | **F-statistic** | **p-value** |
| ***P. damicornis*** | Temperature | 1 | 0.4118403 | 0.41184033 | 1.0153888 | 0.3216741 |
| **Intercellular CA** | CO2 | 2 | 0.1155619 | 0.05778093 | 0.1424584 | 0.8678068 |
|  | Temp*CO2 | 2 | 0.1834858 | 0.09174291 | 0.2261914 | 0.7989133 |

**Supplemental Table 7: Continued**

| **Two-Way ANOVA** | **Effect** | **Df** | **SS** | **Mean Sq** | **F-statistic** | **p-value** |
| --- | --- | --- | --- | --- | --- | --- |
| ***P. damicornis*** | Temperature | 1 | 0.06385155 | 0.063851554 | 2.3530601 | 0.135517 |
| **Extracellular CA** | CO2 | 2 | 0.03881364 | 0.019406819 | 0.7151809 | 0.4972538 |
|  | Temp*CO2 | 2 | 0.01084885 | 0.005424423 | 0.1999011 | 0.8198936 |
|  |  |  |  |  |  |  |
| **Two-Way ANOVA** | **Effect** | **Df** | **SS** | **Mean Sq** | **F-statistic** | **p-value** |
| ***P. damicornis*** | Temperature | 1 | 0.01843521 | 0.018435213 | 1.7717456 | 0.1931941 |
| **CA ATPase** | CO2 | 2 | 0.03628078 | 0.018140392 | 1.7434114 | 0.1921806 |
|  | Temp*CO2 | 2 | 0.01880771 | 0.009403854 | 0.9037724 | 0.4157829 |
|  |  |  |  |  |  |  |
| **Two-Way ANOVA** | **Effect** | **Df** | **SS** | **Mean Sq** | **F-statistic** | **p-value** |
| ***P. damicornis*** | Temperature | 1 | 0.076421 | 0.076421001 | 11.7017899 | **0.001821118** |
| **HSP90** | CO2 | 2 | 0.01357568 | 0.006787838 | 1.039372 | 0.366065225 |
|  | Temp*CO2 | 2 | 0.01243095 | 0.006215476 | 0.9517305 | 0.397421927 |
|  |  |  |  |  |  |  |
| **Two-Way ANOVA** | **Effect** | **Df** | **SS** | **Mean Sq** | **F-statistic** | **p-value** |
| ***P. damicornis*** | Temperature | 1 | 0.05465948 | 0.054659479 | 1.378668 | 0.24956517 |
| **GAPDH** | CO2 | 2 | 0.26228275 | 0.131141373 | 3.307759 | 0.05033163 |
|  | Temp*CO2 | 2 | 0.01917611 | 0.009588053 | 0.241838 | 0.78669929 |

**Supplemental Table 8: Figure 7-Symbiont Gene Expression**

| **Two-Way ANOVA** | **Effect** | **Df** | **SS** | **Mean Sq** | **F-statistic** | **p-value** |  |  |
| --- | --- | --- | --- | --- | --- | --- | --- | --- |
| ***C21a (A. millepora)*** | Temperature | 1 | 0.04456681 | 0.04456681 | 0.2820095 | 0.5995747 |  |  |
| **GLUS** | CO2 | 2 | 0.71703511 | 0.35851755 | 2.2686243 | 0.1221465 |  |  |
|  | Temp*CO2 | 2 | 0.39877013 | 0.19938506 | 1.2616671 | 0.2987891 |  |  |
|  |  |  |  |  |  |  |  |  |
| **Two-Way ANOVA** | **Effect** | **Df** | **SS** | **Mean Sq** | **F-statistic** | **p-value** | **Tukey** |  |
| ***C21a (A. millepora)*** | Temperature | 1 | 0.6622241 | 0.66222405 | 8.950375 | **0.005613962** | LC=MC  MC=HC  LC<HC |  |
| **KETO** | CO2 | 2 | 0.5889634 | 0.29448169 | 3.980105 | **0.029690781** |  |
|  | Temp*CO2 | 2 | 0.2581832 | 0.12909158 | 1.744754 | 0.192528727 |  |
|  |  |  |  |  |  |  |  |  |
| **Two-Way ANOVA** | **Effect** | **Df** | **SS** | **Mean Sq** | **F-statistic** | **p-value** | **Tukey** |  |
| ***C21a (A. millepora)*** | Temperature | 1 | 0.6136346 | 0.6136346 | 4.511619 | **0.04231647** | LC<MC  MC=HC  LC<HC |  |
| **McOA** | CO2 | 2 | 1.4700518 | 0.7350259 | 5.404123 | **0.01011956** |  |
|  | Temp*CO2 | 2 | 0.3206555 | 0.1603278 | 1.178776 | 0.32196578 |  |
|  |  |  |  |  |  |  |  |  |
| **Two-Way ANOVA** | **Effect** | **Df** | **SS** | **Mean Sq** | **F-statistic** | **p-value** |  |  |
| ***C1c-d-t (P. damicornis)*** | Temperature | 1 | 0.4049444 | 0.404944368 | 21.101968 | **7.31921E-05** |  |  |
| **GLUS** | CO2 | 2 | 0.0147693 | 0.007384648 | 0.3848198 | 0.683884368 |  |  |
|  | Temp*CO2 | 2 | 0.0223412 | 0.011170598 | 0.5821086 | 0.564905076 |  |  |
|  |  |  |  |  |  |  |  |  |
| **Two-Way ANOVA** | **Effect** | **Df** | **SS** | **Mean Sq** | **F-statistic** | **p-value** |  |  |
| ***C1c-d-t (P. damicornis)*** | Temperature | 1 | 0.05386393 | 0.053863935 | 6.327121 | 0.017478608 |  |  |
| **KETO** | CO2 | 2 | 0.20535547 | 0.102677736 | 12.061029 | 0.000143286 |  |  |
|  | Temp*CO2 | 2 | 0.09783029 | 0.048915147 | 5.745812 | **0.007716356** |  |  |
|  |  |  |  |  |  |  |  |  |
| **Kruskal-Wallace** | **Effect** | **p-value** | **pair-wise** | HTHC | HTLC | HTMC | LTHC | LTLC |
| ***C1c-d-t (P. damicornis)*** | Temperature | non sig | HTLC | 1 | - | - | - | - |
| **McOA** | CO2 | non sig | HTMC | 0.98 | 1 | - | - | - |
|  |  |  | LTHC | 1 | 1 | 1 | - | - |
|  |  |  | LTLC | 0.97 | 0.91 | 0.68 | 0.92 | - |
|  |  |  | LTMC | 1 | 1 | 0.97 | 1 | 0.98 |

**Supplemental Table 9: SIMPER analysis: Temperature**

|  | **Variables** | **Average Dissimilarity** | **Standard deviation** | **ratio** | **% contribution** |
| --- | --- | --- | --- | --- | --- |
| **A. millepora** | hlipid | 0.02117 | 0.016766 | 1.263 | 13.22 |
|  | hprotein | 0.01902 | 0.017032 | 1.117 | 11.87 |
|  | LEDRr | 0.01814 | 0.013373 | 1.357 | 11.33 |
|  | hcarbo | 0.01739 | 0.016907 | 1.029 | 10.85 |
|  | FvFm | 0.0162 | 0.007785 | 2.082 | 10.12 |
|  | slipid | 0.01597 | 0.014834 | 1.077 | 9.97 |
|  | sprotein | 0.01531 | 0.013281 | 1.153 | 9.56 |
|  | scarbo | 0.01525 | 0.011836 | 1.289 | 9.52 |
|  | PR | 0.01111 | 0.007983 | 1.391 | 6.93 |
|  | volume | 0.01062 | 0.008425 | 1.261 | 6.63 |
|  |  |  |  |  |  |
| **P. damicornis** | LEDRr | 0.019431 | 0.012869 | 1.51 | 13.59 |
|  | hlipid | 0.018664 | 0.014081 | 1.325 | 13.06 |
|  | slipid | 0.018622 | 0.017224 | 1.081 | 13.03 |
|  | scarbo | 0.017283 | 0.012975 | 1.332 | 12.09 |
|  | hcarbo | 0.015662 | 0.013333 | 1.175 | 10.96 |
|  | sprotein | 0.01317 | 0.011131 | 1.183 | 9.21 |
|  | hprotein | 0.012723 | 0.009209 | 1.382 | 8.9 |
|  | PR | 0.011775 | 0.009695 | 1.215 | 8.24 |
|  | volume | 0.008777 | 0.006336 | 1.385 | 6.14 |
|  | FvFm | 0.006835 | 0.003311 | 2.064 | 4.78 |

**Supplemental Table 9: SIMPER analysis: Temperature continued**

|  |  |  |  |  |  |
| --- | --- | --- | --- | --- | --- |
| **M. monasteriata** | slipid | 0.030115 | 0.02437 | 1.236 | 16.64 |
|  | LEDRr | 0.025096 | 0.016328 | 1.537 | 13.87 |
|  | hcarbo | 0.020284 | 0.01667 | 1.217 | 11.21 |
|  | hprotein | 0.019484 | 0.013287 | 1.466 | 10.77 |
|  | hlipid | 0.018015 | 0.018001 | 1.001 | 9.96 |
|  | scarbo | 0.015297 | 0.013114 | 1.166 | 8.45 |
|  | volume | 0.015076 | 0.010452 | 1.442 | 8.33 |
|  | PR | 0.015075 | 0.011736 | 1.285 | 8.33 |
|  | sprotein | 0.014846 | 0.012803 | 1.16 | 8.21 |
|  | FvFm | 0.007659 | 0.005412 | 1.415 | 4.23 |
|  |  |  |  |  |  |
| **T. reniformis** | sprotein | 0.032555 | 0.023252 | 1.4001 | 15.67 |
|  | slipid | 0.032457 | 0.022394 | 1.4493 | 15.63 |
|  | LEDRr | 0.026393 | 0.018766 | 1.4064 | 12.71 |
|  | hcarbo | 0.022442 | 0.018231 | 1.231 | 10.81 |
|  | scarbo | 0.021306 | 0.017366 | 1.2269 | 10.25 |
|  | hlipid | 0.020377 | 0.02279 | 0.8941 | 9.82 |
|  | hprotein | 0.018 | 0.014965 | 1.2028 | 8.66 |
|  | volume | 0.015385 | 0.009836 | 1.5642 | 7.41 |
|  | PR | 0.012941 | 0.016865 | 0.7674 | 6.23 |
|  | FvFm | 0.005834 | 0.004211 | 1.3855 | 2.81 |
